# Functional Analysis of the Cathepsin-Like Cysteine Protease Genes in Adult *Brugia malayi* Using RNA Interference

**DOI:** 10.1371/journal.pntd.0000377

**Published:** 2009-02-10

**Authors:** Louise Ford, Jun Zhang, Jing Liu, Sarwar Hashmi, Juliet A. Fuhrman, Yelena Oksov, Sara Lustigman

**Affiliations:** 1 Laboratory of Molecular Parasitology, Lindsley F. Kimball Research Institute, New York Blood Center, New York, New York, United States of America; 2 Department of Biology, Tufts University, Medford, Massachusetts, United States of America; 3 Laboratory of Electron Microscopy, Lindsley F. Kimball Research Institute, New York Blood Center, New York, New York, United States of America; George Washington University Medical Center, United States of America

## Abstract

**Background:**

Cathepsin-like enzymes have been identified as potential targets for drug or vaccine development in many parasites, as their functions appear to be essential in a variety of important biological processes within the host, such as molting, cuticle remodeling, embryogenesis, feeding and immune evasion. Functional analysis of *Caenorhabditis elegans* cathepsin L (*Ce-cpl-1*) and cathepsin Z (*Ce-cpz-1*) has established that both genes are required for early embryogenesis, with *Ce-cpl-1* having a role in regulating in part the processing of yolk proteins. *Ce-cpz-1* also has an important role during molting.

**Methods and Findings:**

RNA interference assays have allowed us to verify whether the functions of the orthologous filarial genes in *Brugia malayi* adult female worms are similar. Treatment of *B. malayi* adult female worms with *Bm-cpl-1*, *Bm-cpl-5*, which belong to group Ia of the filarial *cpl* gene family, or *Bm-cpz-1* dsRNA resulted in decreased numbers of secreted microfilariae *in vitro*. In addition, analysis of the intrauterine progeny of the *Bm-cpl-5* or *Bm-cpl Pro* dsRNA- and siRNA-treated worms revealed a clear disruption in the process of embryogenesis resulting in structural abnormalities in embryos and a varied differential development of embryonic stages.

**Conclusions:**

Our studies suggest that these filarial cathepsin-like cysteine proteases are likely to be functional orthologs of the *C. elegans* genes. This functional conservation may thus allow for a more thorough investigation of their distinct functions and their development as potential drug targets.

## Author Summary

Filarial nematodes are an important group of human pathogens, causing lymphatic filariasis and onchocerciasis, and infecting around 150 million people throughout the tropics with more than 1.5 billion at risk of infection. Control of filariasis currently relies on mass drug administration (MDA) programs using drugs which principally target the microfilarial life-cycle stage. These control programs are facing major challenges, including the absence of a drug with macrofilaricidal or permanent sterilizing activity, and the possibility of the development of drug-resistance against the drugs available. Cysteine proteases are essential enzymes which play important roles in a wide range of cellular processes, and the cathepsin-like cysteine proteases have been identified as potential targets for drug or vaccine development in many parasites. Here we have studied the function of several of the cathepsin-like enzymes in the filarial nematode, *B. malayi*, and demonstrate that these cysteine proteases are involved in the development of embryos, show similar functions to their counterparts in *C. elegans*, and therefore, provide an important target for future drug development targeted to eliminate filariasis.

## Introduction

Human lymphatic filariasis (LF), caused by the filarial parasites *Brugia malayi*, *Brugia timori* and *Wuchereria bancrofti*, infects 120 million people worldwide, of which 40 million people show chronic disease symptoms (www.globalnetwork.org) [1]. The disease is estimated to be responsible for 5.5 million DALYs, and is the second leading cause of permanent and long-term disability worldwide [2]. A further one billion people (18% of the world's population) are at risk of infection (www.globalnetwork.org).

The Global Programme to Eliminate Lymphatic Filariasis (GPELF) aims to use mass drug administration (MDA) to interrupt transmission and to reduce morbidity, with annual doses of a multi-drug regimen for at least five years (www.filariasis.org) [3]. The African Programme for Onchocerciasis Control aims to establish by 2010 community-based sustainable ivermectin treatments of 50 million people in 19 African countries having meso- and hyper-endemicity (www.who.int/apoc/en/). However, it appears unlikely that any of these MDA regimens will be sufficient to eliminate LF or onchocerciasis in all endemic areas [2]. Numerous technical challenges threaten the success of these eradication programs [2],[4], including incomplete efficacy of available drugs against adult filarial worms [5],[6], severe drug toxicity in people with heavy loiasis infections [3], and the risk that filarial worms will develop resistance to the drugs available for MDA [7],[8]. Ivermectin (IVM) resistance has been reported worldwide in several other parasitic nematodes [9].

The emergence of drug resistant strains of the *O. volvulus* parasite was initially suggested by reports of patients with onchocerciasis who failed to respond to IVM treatment [10],[11] and a recent report from Ghana has provided the first direct proof of IVM resistance in *O. volvulus* populations [12]. A number of studies have associated IVM resistance with genetic markers (reviewed in [13]), while a recent, more extensive study using worms taken from individuals before and after repeated IVM treatments, has demonstrated that IVM causes genetic selection on *O. volvulus* [14]. At present there are no alternative drugs for IVM for use in the onchocerciasis MDA programs that can reduce Mf or kill adult worms and no vaccines are available [15]. Vaccines that decrease transmission would complement MDA programs and may be necessary for complete elimination [13]. Notably, antibiotic treatment targeting *Wolbachia* endosymbionts of filarial nematodes is emerging as an alternative drug treatment for filariasis. Doxycycline treatment has been shown to result in an almost complete loss of Mf and macrofilaricidal effects in LF and in sterilization of adult worms and macrofilaricidal effects in *O. volvulus* (reviewed in [16],[17]). However, the period of treatment with doxycycline at present (between 4–8 weeks) is not applicable to mass treatment strategies, due to both the logistical difficulties and contra-indication in children under eight and pregnant women [16],[17], as a result antibiotic treatment regimens require further refinement.

Therefore, a major priority has to be the identification of new drugs with strong activity against adult filarial worms (macrofilaricidal) which have new classes of chemistry, new molecular targets, and novel modes of action. Recent breakthroughs in genomics and chemistry make macrofilaricidal drug development more feasible, and accordingly a high priority goal with the WHO/TDR and the Bill & Melinda Gates Foundation [8],[18],[19],[20],[21].

RNA interference (RNAi) was first described in *Caenorhabditis elegans* where it was shown to spread systemically throughout the whole organism [22] and is widely used to identify gene function and has been developed for high-throughput genomics [23],[24],[25]. This powerful reverse genetics mechanism thus provides an invaluable tool which could be transferred to gene function studies and novel drug discovery in filarial nematodes. Moreover, it potentially provides an unprecedented opportunity to identify pre-validated drug targets after efficient mining of nematode genomic databases and RNAi genome wide *C. elegans* databases. [26],[27] [28] [29]. RNAi has been successfully demonstrated in a number of parasitic nematodes (reviewed in [30],[31]), including filarial nematodes [32],[33],[34],[35],[36]. However, although RNAi has been demonstrated in parasitic nematodes, its application as a tool for high-throughput functional genomic screening for identification of essential parasite genes has not yet been achieved due to variable success in transferring the technology from *C. elegans* to parasitic nematodes and problems with inconsistent results and poor reproducibility [30],[37].

While a wide range of target genes have been screened using RNAi in parasitic nematodes, only a small number of these genes have been selected due to their potential as drug targets [31]. Filarial proteases have been recognized as potential drug targets [33],[38]. RNAi targeting the cathepsin L- and Z-like cysteine proteases (CPL & CPZ respectively) has clearly validated their essential role during *O. volvulus* L3 molting [33]. The function(s) of CPLs in filarial nematodes during embryogenesis, however, was only predicted indirectly by immunoelectron microscopy (IEM) [39],[40], while detailed studies of the CPLs in *C. elegans* [40],[41] have provided more direct proof of their function in the free living nematodes. However, their predicted essential function during development may not always be conserved in both filarial nematodes and *C. elegans*, as has been shown for the roles of CPL and CPZ in the molting of *O. volvulus* and *C. elegans* [33]. Therefore it would be beneficial to be able to directly and reliably assess their essential function(s) using RNAi technology in filarial adult worms, in particular during embryogenesis. Here we demonstrate the use of double-stranded RNA (dsRNA)-mediated silencing to study the possible function of several of the cathepsin-like enzymes in *B. malayi*.

## Materials and Methods

### Parasites

Adult female *B. malayi*, collected from the peritoneal cavities of infected jirds (*Meriones unguiculatus*) at day 80–85 post-infection, were kindly provided by the NIAID/NIH Filariasis Research Reagent Repository Center (Athens, GA; www.filariasiscenter.org). Worms were washed once in normal culture medium (CM; RPMI-1640, 100 U/ml penicillin, 100 µg/ml streptomycin, 2 mM L-glutamine, 2.5 µg/ml amphotericin B, and 25 mM HEPES (Sigma, St Louis, MO)) preheated to 37°C. Individual worms were transferred into 1 ml of normal CM in 48 well culture plates (Corning Inc. Life Sciences, Lowell, MA) and cultured overnight at 37°C under 5% CO_2_ to ensure the absence of any contaminating microorganisms. Release of Mf was measured after overnight culture. Viable and motile worms, which were secreting motile Mf and were in the middle of the Mf release distribution, were selected for RNAi treatment.

### Cloning of target *B. malayi* gene fragments for *in vitro* transcription

The GenBank accession numbers of the targeted *B. malayi* cathepsin-like cysteine protease (CP) genes and the gene-specific primers used to amplify the target genes are listed in Table 1. Fragments corresponding to cDNA regions of the *B. malayi* cathepsin-like genes; *Bm-cpl-1*, *Bm-cpl-5*, and *Bm-cpz*, were amplified by PCR using gene-specific primers designed against the published sequences [39] (Table 1). A smaller fragment corresponding to pro-region of the *Bm-cpl* genes (*Bm-cpl Pro*), designed to knock-down the three *B. malayi cpl* genes; *Bm-cpl-1*, *Bm-cpl-4* and *Bm-cpl-5* (group Ia of the filarial cathepsin L-like cysteine protease gene family [39]), which have identical pro-region sequences, was amplified using gene-specific primers (Table 1). A fragment corresponding to an intronic sequence within intron 2 of the *O. volvulus cpz* gene sequence *Ov-cpz-int2* (position 621-1132, GenBank accession no. AY591516), was amplified as a negative control as described previously [33]. The sequence was checked for putative microRNAs (miRNA) using the miRBase database (http://microrna.sanger.ac.uk/sequences/index.shtml) and by folding it using Mfold (http://frontend.bioinfo.rpi.edu/applications/mfold/cgi-bin/rna-form1.cgi). These analyses were used to determine if *Ov-cpz-int2* contains any strong hairpins with a delta G≤−25 kcal/mol which might indicate a unique miRNA which could potentially initiate miRNA silencing at the translational level [42],[43]. Both methods have shown that the *Ov-cpz-int2* sequence has no miRNA sequences (C. Poole&L. McReynolds, New England Biolabs, personal communication) that could have induced silencing of the target gene using a different pathway to dsRNA-induced silencing. *E. coli* β-lactamase (*Ec-bla*) dsRNA (GenBank accession no. NC_010862, position 24517-24770) [31] was also used as a negative control. The PCR fragments were then sub-cloned into pCR4-TOPO vector (Invitrogen, Carlsbad, CA) or pBluescript SK vector (Stratagene, La Jolla, CA) according to the manufacturer's instructions. Clones were confirmed by sequencing both strands. Plasmids were then purified using the Rapid Plasmid Miniprep System (Marligen Biosciences Inc., Ijamsville, MD) and were used as templates for RNA *in vitro* transcription.

**Table 1:** GenBank accession numbers, and primer sequences and positions used for gene specific dsRNA production and real-time RT-PCR (qRT-PCR) with the resulting fragment size.

Gene target	Accession number	Primer name	Position	Primers (5′-3′)	Gene specific primer product
		**dsRNA production**			
					
*Bm-cpl-1*	AF331035	Bm-cpl-1F	548	CCAAGTTCAGTTGATTGG	642 bp
		Bm-cpl-1R	1189	GATGGGAAATGAACCCATCG	
*Bm-cpl-5*	AY533167	Bm-cpl-5F	550	CTGCCAGATCAAGTTGAC	648 bp
		Bm-cpl-5R	1197	CGGGAAATGAAGCCATAG	
*Bm-cpl Pro*	AY533167	Bm-cpl Pro F	117	CAATATGACGAGACTTGCGT	334 bp
		Bm-cpl Pro R	450	GACCATAAATTCTTCATCGG	
*Bm-cpz*	AY533170	Bm-cpz F	200	CGAAGACTTACCTATAGC	289 bp
		Bm-cpz R	488	TTTCGTGTGGTATGCCAAC	
*Ov-cpz-int2*	AY591516	Int2 F	621	TTCCTTTTCGGAGAATTAGC	512 bp
		Int2 R	1132	TATGGAAAAAGATCGAAATT	
		**qRT-PCR**			
*Bm-tub-1*	AY705382	Bm-tubF	173	AATATGTGCCACGAGCAGTC	307 bp
		Bm-tubR	479	GGATACTCCTCACGAATTT	
*Bm-cpl*	AY533167	Bm-cpl RTF	100	GACAAAGATTACAAACAGGGC	381 bp
		Bm-cpl RTR	480	TGATTGGGCAGTCGAAGTC	

### Production of dsRNA, siRNA and Cy3-labeled RNAs

For RNA transcription, cDNA template from the purified plasmid was amplified with M13 forward and M13 reverse primers (Invitrogen) and then used with either T3 and T7 RNA polymerase for the single-stranded sense or antisense RNA synthesis using the MEGAscript high yield transcription kit (Ambion Inc., Austin, TX). Large quantities of dsRNA were prepared as previously described [44]. Integrity of dsRNA was checked by standard agarose gel electrophoresis. The final size (bp) of dsRNA is approximately 120 bp larger than the amplified gene-specific fragment due to vector linker sequence between the T3 and T7 priming sites and inserted gene fragment. Short-interfering RNA (siRNA) corresponding to the specific target was produced by digesting transcribed dsRNA with RNase III (Ambion) according to the manufacturer's instructions. Undigested and partially RNaseIII digested material was removed using a siRNA purification unit (Ambion). The siRNA was quantified by measuring absorbance and the concentration calculated according to the manufacturer's instructions.

The penetration of both dsRNA and siRNA into adult female *B. malayi* was followed using fluorescently-labeled RNA. dsRNA and siRNA were fluorescently labeled with cyber red, Cy3 (Cy3-RNA), using the Silencer siRNA labeling kit (Ambion) according to the manufacturer's instructions. *B. malayi* adult female worms, in groups of 4, were soaked in normal CM containing Cy3-RNA (0.01 mg/ml) for 24–72 h. Fluorescence was visualized using a Zeiss Axiovert fluorescence microscope using the rhodamine filter set, using emission 590 nm.

### RNA interference (RNAi) treatment of *B. malayi* adult females

RNAi treatment of *B. malayi* adult females was carried out by soaking with dsRNA or siRNA. dsRNA preparations were dialyzed for 3 h in D-tube Maxi dialysis tubes, 12–14 kDa cut-off (Novagen, EMD Biosciences, Inc., Madison, WI), at 37°C under 5% CO_2_, against normal culture medium before incubation with *B. malayi* adult female worms.

Following 24 h culture in normal culture medium at 37°C in a 5% CO_2_ incubator, viable and motile worms were transferred into 48-well plates in groups of 3–4 adult females per well. Each group was cultured for upto 3 d in 1 ml of normal CM containing 1.5–2 mg/ml dsRNA or 5 µM (45 µg/ml) siRNA, with a daily change of media containing dsRNA. Following RNAi treatment, worms were transferred to normal CM containing 10% heat-inactivated fetal calf serum and cultured for an additional 2 d, with daily change of media. Every 24 h throughout the experiment adult female phenotypes were monitored microscopically, and the number and the phenotype of the secreted progeny, microfilariae (Mf), pre-microfilariae (p-mf), embryos and eggs, were recorded. To compare progeny release after RNAi treatment, release was expressed as a reduction in release in comparison to the release in the dsRNA-free medium control group. At the end of the experiment (2 d after RNAi treatment) worms were collected for either embrograms, where intrauterine progeny were recovered from individual female worms, or RNA extraction for quantitative real-time PCR (qRT-PCR) analysis. Worms were gently homogenized in 0.5 ml normal culture medium for 2–3 min, and the resulting suspension was examined microscopically to determine the relative proportions of progeny at different stages of development.

RNAi treatment with dsRNA was repeated using dialysis tubes instead of 48-well plates, as previously demonstrated by Aboobaker *et al* (2003) [34] using a modified protocol. Worms were incubated in dsRNA for 4 d in dialysis tubes (D-tube Maxi dialysis tubes, 12–14 kDa cut-off) and dialyzed against normal culture medium, which allowed for a longer incubation time in dsRNA and no further handling of the worms during the incubation period.

*Bm-cpl-1*, *Bm-cpl-5*, *Bm-cpl Pro* and *Bm-cpz* dsRNAs were used as the test RNAi treatments, with negative controls being either culture medium containing RNA storage buffer (medium control), dsRNA corresponding to an *O. volvulus* intronic sequence *Ov-cpz-int2* or *E. coli* β-lactamase (*Ec-bla*) (negative control). Each experiment was repeated at least three times.

### Real-time RT-PCR

Loss of specific transcripts following RNAi treatment were examined by real-time quantitative RT-PCR (qRT-PCR). Primers used were designed to prevent re-amplification from dsRNA (Table 1) and to amplify all three of the *B. malayi* filarial group Ia cathepsin L-like cysteine protease genes; *Bm-cpl-1*, *Bm-cpl-4* and *Bm-cpl-5* (*Bm-cpl*). Our initial attempts to design dsRNA-mediated silencing confirmation primers to distinguish the three *Bm-cpl* genes were unsuccessful; *Bm-cpl-4* and *Bm-cpl-5* are 94% identical, while *Bm-cpl-1* is 83% identical to *Bm-cpl-4* and *Bm-cpl-5*. Moreover, because of the strong similarity, we assumed that dsRNA-mediated silencing with *Bm-cpl-5* dsRNA will cross-target also *Bm-cpl-4* and *Bm-cpl-1*. However, it will not be able to cross-target the other five phylogenetically distinct *cpl* genes that belong to group Ic [39]. This was further established by comparing the predicted siRNA in these three transcripts to those of group Ic using the Protein Lounge siRNA Database (www.proteinlounge.com); none of which were similar to those predicted for *Bm-cpl-1*, *-4* and *-5*. *B. malayi* β-tubulin (*Bm-tub-1*; GenBank accession no. AY705382) was used as the endogenous control gene. At the end of the experiment (2 d after RNAi treatment), groups of four adult female worms were removed from culture, washed in PBS, and flash frozen in liquid N_2_. Frozen worms were homogenized in Trizol reagent (Invitrogen) and RNA was extracted as previously described [40]. First strand cDNA was generated using the SuperScript III first strand cDNA synthesis kit (Invitrogen) and priming with oligo(dT)20. The specific cDNA fragments were then amplified by real-time PCR using QuantiTect SYBR Green PCR kit (Qiagen Inc, Valencia, CA) and the ABI Prism 7700 Sequence Detection System (Applied Biosystems, Foster City, CA). The PCR conditions used were 50°C for 2 min, 95°C for 15 min, followed by 40 cycles of 94°C for 15 s, 52–58°C for 30 s, 72°C for 30 s. The relative amount of test amplicon in each experiment was determined by using the comparative *C_T_* method normalizing against the endogenous control gene (*Bm-tub-1*), as described in the ABI PRISM Sequence Detection System User Bulletin No2 (Applied Biosystems). The value of the medium control group was set to 100% and the relative reduction of the RNAi treated groups was calculated and expressed as a percentage reduction in comparison to the control group.

### Statistical Analysis

Comparison between the groups in RNA interference experiments were analyzed using the two-tailed non-parametric Mann-Whitney *U*-test. A *P* value of <0.05 was considered statistically significant.

## Results

### dsRNA-mediated silencing of the *B. malayi* cathepsin-like cysteine protease genes, *Bm-cpl-1*, *Bm-cpl-5*, and *Bm-cpz* results in the reduction of Mf secretion *in vitro*

**Figure 1:**
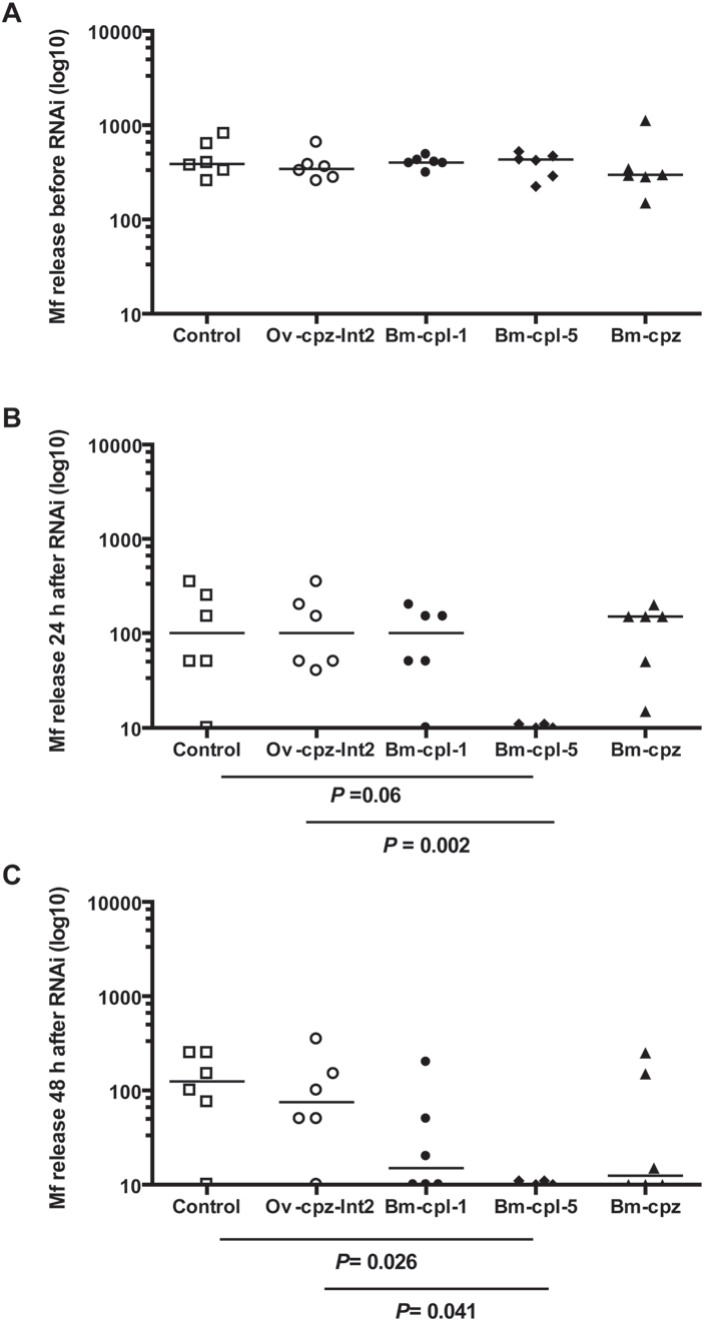
dsRNA-mediated silencing of the *B. malayi* cathepsin-like genes, *Bm-cpl-1*, *Bm-cpl-5*, and *Bm-cpz* leads to a reduction in microfilaria release from *B. malayi in vitro*. Following 24 h culture in normal culture medium, two groups of 3 female *B. malayi* worms were soaked for 18 h in 2 mg/ml gene-specific dsRNA (*Bm-cpl-1*, *Bm-cpl-5*, *Bm-cpz*, *Ov-cpz-Int2*) or medium alone (control). Following dsRNA treatment, individual worms were transferred to dsRNA-free medium and cultured for an additional 48 h. Released microfilariae were collected and counted daily. Results are expressed as Mf release before RNAi treatment (A), 24 h (B) and 48 h (C) after treatment. Each graph represents one experiment which is representative of at least 3 separate experiments. *P* values denote a significant difference between dsRNA-treated worms and either untreated medium controls or negative control (*Ov-cpz-Int2*) (Mann-Whitney *U*-test).

To determine the possible function(s) of the *B. malayi* cathepsin-like cysteine protease genes during embryogenesis of *B. malayi*, RNAi was carried out to selectively interfere with *Bm* cathepsin-like cysteine protease gene expression; *Bm-cpz* or *Bm-cpl*. Microfilarial release was determined prior to RNAi treatment to provide a baseline reading and shows that grouped worms were evenly distributed in terms of Mf release (Fig. 1A). Daily microfilarial release was determined from control and RNAi treated adult female worms which were cultured for 18 h in the presence of gene-specific dsRNA (2 mg/ml) and then cultured for an additional 48 h in dsRNA-free culture medium. In comparison to the control worms and the worms treated with a negative control dsRNA (*Ov-cpz-int2*), adult worms treated with dsRNAs corresponding to the cathepsin-like cysteine protease genes, *Bm-cpl-1*, *Bm-cpl-5* and *Bm-cpz*, all showed a reduction in the release of Mf into the culture medium (Fig. 1B & C). This reduction in the secretion of Mf corresponded to a 71.4% (*Bm-cpl-1*), 92.8% (*Bm-cpl-5*) and 61.3% (*Bm-cpz*), inhibition (average) of Mf release 48 h after RNAi treatment. The reduction in the release of Mf after treatment with *Bm-cpl-5* dsRNA was significantly different from both the control worms (*P* = 0.026; 48 h after RNAi) and the negative control (*Ov-cpz-int2*) treated worms (*P* = 0.002 and 0.041; 24 h and 48 h after RNAi respectively) (Fig. 1). This demonstrates a persistent effect of dsRNA-mediated silencing as worms had been cultured in dsRNA-free medium for a further 24–48 h following RNAi treatment. As we saw the most significant reductions in the release of Mf after treatment with dsRNA corresponding to *Bm-cpl-5* (Fig. 1B & C) we decided to focus on optimization of the RNAi technique and analyzing the effect on embryogenesis using dsRNA corresponding to *Bm-cpl-5* and to the identical pro-region of *Bm-cpl-1*, *Bm-cpl-4* and *Bm-cpl-5*; *Bm-cpl Pro*, which was designed to target all three *Bm-cpl* transcripts.

### Uptake of Cy3-labeled RNAs by adult female *B. malayi*

In order to optimize and utilize the RNAi soaking technique in *B. malayi* to target the cathepsin-like cysteine protease genes it was important to demonstrate uptake of the *in vitro* transcribed dsRNA and siRNA by adult female *B. malayi*. FITC-labeled *Bm-tub-1* dsRNA (approx. 300 bp) has previously been shown to be taken up successfully by adult female *B. malayi* after soaking for 18 h with 0.08 mg/ml dsRNA [34].

**Figure 2:**
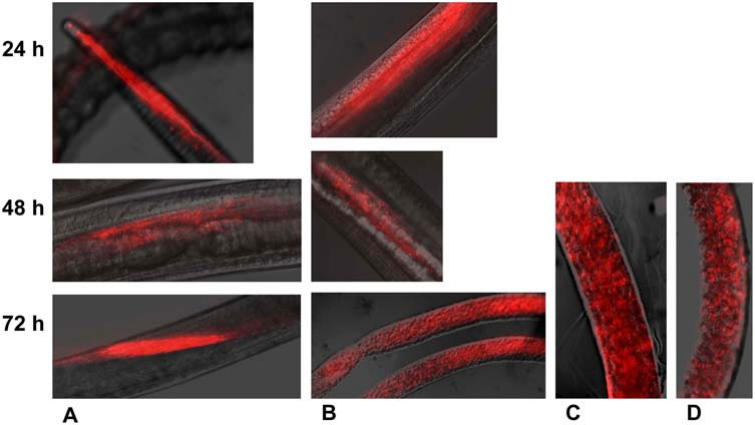
Demonstration of uptake of Cy3-labeled dsRNAs or siRNA by *B. malayi*. Adult female *B. malayi* (2 groups of 4 worms) were soaked in normal culture medium containing Cy3-dsRNA (0.01 mg/ml) for 24–72 h. Uptake was examined for *Bm-cpl-5* dsRNA (∼800 bp) (A), *Bm-cpl-5* siRNA (B), *Bm-cpl Pro* dsRNA (∼400 bp) (C) and *Bm-cpl Pro* siRNA (D). Fluorescence was visualized using a Zeiss Axiovert fluorescence microscope using the rhodamine filter set, using emission 590 nm.

Cy3-labeled *Bm-cpl* RNAs, both dsRNA and siRNA, were taken up by adult worms *in vitro* after soaking for 24–72 h at 0.01 mg/ml (Fig. 2). Uptake of Cy3-labeled RNA was clearly seen after 24 h incubation with Cy3-labeled RNAs corresponding to a fragment of *Bm-cpl-5*. The dsRNA *Bm-cpl-5* fragment appeared in the mouth, esophagus and intestine after 24 h and in the cuticular and hypodermal regions after 48 h and 72 h (Fig. 2A). Uptake of the Cy3-labeled RNAs appeared to be affected by the size of the dsRNA. While the dsRNA *Bm-cpl-5* fragment (approx. 800 bp) was taken up by the adult worms and was seen in the hypodermal regions only after 48 h, the siRNA appeared in these regions earlier and was clearly already seen after 24 h (Fig. 2B). In addition, a smaller dsRNA corresponding to the pro-region of *Bm-cpl*; *Bm-cpl Pro* (approx. 400 bp) gave a more intense and diffuse uptake staining pattern after 72 h when compared to the uptake of the larger *Bm-cpl-5* fragment (Fig. 2C). The smaller *Bm-cpl Pro* Cy3-labeled dsRNA and the Cy3-labeled siRNAs were also found along the length of the uterus. It is important to note that while the dsRNAs were added at the same concentration (wt/vol), they have different molarities due to their different lengths, *Bm-cpl-5*; approx. 800 bp, 0.019 µM, *Bm-cpl Pro*; approx. 400 bp, 0.038 µM, siRNA; approx 13.5 bp, 1.1 µM. This uptake was consistent in all worms examined.

We also examined whether the use of a lipid carrier (lipofectin) could improve the uptake of dsRNA. Lipofectin was not toxic to *B. malayi* adult female worms, however, lipofectin mixed with *Bm-cpl-5* dsRNA did not improve the penetration of dsRNA (data not shown).

### dsRNA-mediated silencing of *B. malayi cpl-5* and the pro-region of *Bm-cpl* results in the inhibition of Mf secretion *in vitro*, death of Mf, and changes in intrauterine progeny potentially resulting from a specific change in *Bm-cpl* transcript level

**Figure 3:**
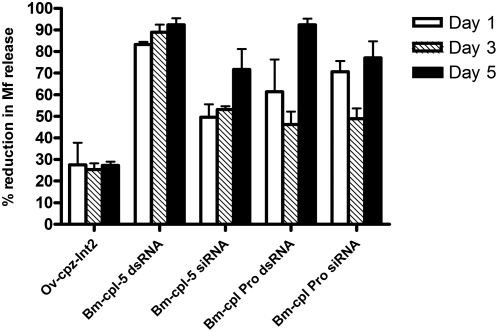
dsRNA treatment of adult female *B. malayi* with *Bm-cpl-5* and the pro-region of *Bm-cpl* (*Bm-cpl Pro*) leads to an inhibition of *B. malayi* microfilaria secretion *in vitro*. 8 female worms (2 groups of 4 worms) were treated for 3 d with 1.5 mg/ml gene-specific dsRNA or 5 µM siRNA, corresponding to *Bm-cpl-5* or *Bm-cpl-Pro*. Worms were then cultured for a further 2 d in culture medium alone. Mf secretion was recorded daily and release expressed as a reduction in release in comparison to the dsRNA-free medium control group on day 1 (open bars), day 3 (hashed bars), and day 5 (closed bars). Data represents one representative experiment from at least 3 separate experiments.

We next examined in more detail the effect of RNAi selectively carried out to interfere with *Bm-cpl* gene expression using both *Bm-cpl-5* and *Bm-cpl Pro*. Microfilarial release was determined daily from worms cultured in the presence of gene-specific dsRNA (1.5 mg/ml, *Bm-cpl-5*; 2.82 µM, *Bm-cpl Pro*; 5.63 µM) or their corresponding siRNA (5 µM) for 3 d, and then cultured for a further 2 d in dsRNA-free culture medium. In comparison to the control worms, adult worms treated with dsRNA or siRNA corresponding to the *Bm-cpl* genes showed a persistent and significant reduction in the release of Mf into the culture medium after 1, 3 and 5 days (*P*<0.05 for all) (Fig. 3). Reduction in Mf release after treatment with *Bm-cpl-5* dsRNA was equivalent to that shown in Fig. 1. Although treatment with the negative control dsRNA, *Ov-cpz-int2*, did result in a reduction in the release of Mf this reduction was not significantly different from the medium control (*P*>0.05) (Fig. 3). Reduction in the release of Mf was seen most rapidly after treatment with *Bm-cpl-5* dsRNA, however, by day 5 both *Bm-cpl-5* and *Bm-cpl Pro* dsRNA treatment had equivalent reductions in Mf release. Importantly, reductions in Mf release were also seen after treatment with siRNA corresponding to the *Bm-cpl-5* and *Bm-cpl Pro* genes (Fig. 3). We also noted that in the groups treated with either *Bm-cpl-5* or *Bm-cpl Pro* dsRNA the majority of Mf which were released from day 1 onwards were immotile and granulated (approx. 90–95%) suggesting that the Mf were dead (data not shown).

**Figure 4:**
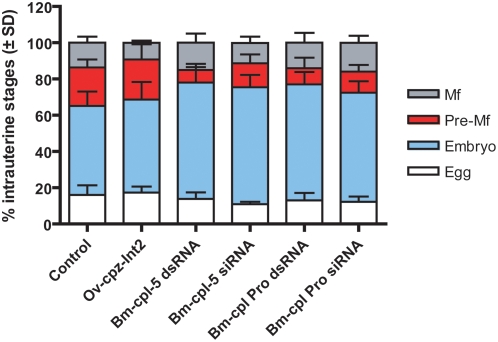
Embryogenesis effects following treatment of adult female *B. malayi* with dsRNA or siRNA. Intrauterine progeny from individual female worms (groups of four adult worms) were examined 2 d after dsRNA treatment and expressed as the relative proportions of progeny at different stages of development. Data represents one representative experiment from at least 3 separate experiments.

**Figure 5:**
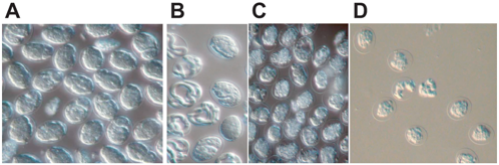
Treatment of adult female *B. malayi* with *Bm-cpl-1*, *Bm-cpl-5*, and *Bm-cpz* dsRNAs leads to phenotypic changes in developing embryos. Intrauterine progeny from individual female worms were examined 2 d after treatment with medium control (A), *Ov-cpz-Int2* control dsRNA (B), *Bm-cpl-5* dsRNA (C) and *Bm-cpl Pro* dsRNA (D).

RNAi treatment with *Bm-cpl-5* and *Bm-cpl Pro* genes had dramatic effects on intrauterine embryogram profiles (Fig. 4). Intrauterine progeny were examined 2 d after RNAi treatment and expressed as the relative proportions of progeny at different stages of development; eggs, developing embryos, pre-microfilariae (pre-Mf) and Mf. In comparison to the percentage of intrauterine pre-Mf in both the medium control (21.2%) and the negative control, *Ov-cpz-int2* (22.1%) adult female worms treated with *Bm-cpl-5* and *Bm-cpl Pro* dsRNA or siRNA showed significant reductions in pre-microfilariae (pre-Mf); *Bm-cpl-5* dsRNA; 6.8% (Control; *P* = 0.002, *Ov-cpz-int2*; *P* = 0.004), *Bm-cpl-5* siRNA; 13.2% (Control; *P* = 0.0037, *Ov-cpz-int2*; ns), *Bm-cpl Pro* dsRNA; 9.0% (Control; *P* = 0.0011, *Ov-cpz-int2*; *P* = 0.028), and *Bm-cpl Pro* siRNA; 11.6% (Control; *P* = 0.0003, *Ov-cpz-int2*; *P* = 0.016). While the numbers of pre-Mf were reduced, the percentage of embryos within the uterine progeny were significantly increased in comparison to medium control (49.1%) and the negative control, *Ov-cpz-int2* (51.3%); *Bm-cpl-5* dsRNA; 64.2% (Control; *P* = 0.002, *Ov-cpz-int2*; *P* = 0.048), *Bm-cpl-5* siRNA; 64.5% (Control; *P* = 0.0037, *Ov-cpz-int2*; *P* = 0.042), *Bm-cpl Pro* dsRNA; 64.0% (Control; *P* = 0.0011, *Ov-cpz-int2*; *P* = 0.028), and *Bm-cpl Pro* siRNA; 60.2% (Control; *P* = 0.0003, *Ov-cpz-int2*; ns). No changes were observed in the proportions of Mf or eggs within the uterus. Phenotypic changes and structural abnormalities in developing embryos were also observed following RNAi treatment (Fig. 5). Treatment with either *Bm-cpl-5* dsRNA (Fig. 5C) or *Bm-cpl Pro* dsRNA (Fig. 5D) resulted in malformed intrauterine embryos in comparison to both the medium control (Fig. 5A) and negative control (Fig. 5B). Embryos from treated worms appeared to be not fully developed within the eggshell, leading to space between the embryo and eggshell. Effects on embryonic viability were also observed following RNAi treatment with *Bm-cpl* suggesting that the eggs and embryos released from *Bm-cpl* treated *B. malayi* were less viable (18–22% viable) than those from controls as demonstrated by MTT viability staining [45] (data not shown).

RNAi treatment was repeated using dialysis tubes instead of the culture plate system. Worms were incubated in dsRNA for 4 d in dialysis tubes and dialyzed against normal culture medium, which allowed for a longer incubation time in dsRNA with the total amount of dsRNA required being much less. RNAi treatment targeting the *B. malayi* cathepsin-like cysteine protease group Ia genes using dialysis tubes resulted in similar phenotypic effects to those seen in the culture plate system (data not shown). RNAi in dialysis tubes has the potential to facilitate higher-throughput RNAi screens for selected genes.

**Figure 6:**
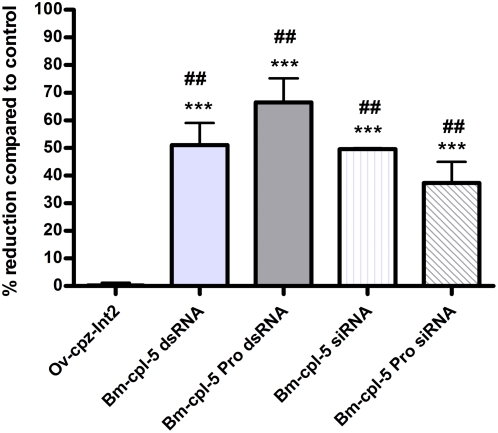
Soaking *B. malayi* adult female worms in dsRNA results in *Bm-cpl* gene-specific inhibition of expression. At the end of the experiment (2 d after treatment), groups of four adult female worms were removed from culture and analyzed for differences in *Bm-cpl* gene-specific transcript levels by qRT-PCR. The relative amounts of *Bm-cpl* amplicon were determined by using the comparative *C_T_* method and normalizing against the endogenous control gene (*Bm-tub-1*). The median value of the control group was set to 100% and the reduction in expression in the treated groups was calculated as a percentage of the control. ## denotes a significant difference between dsRNA or siRNA-treated worms and medium control, while *** denotes a significant difference between dsRNA or siRNA-treated worms and negative control (dsRNA of *Ov-cpz-Int2*) (Mann-Whitney *U*-test).

RNAi treatment targeting *Bm-cpl-5* and *Bm-cpl Pro* using both dsRNA and siRNA resulted in a specific reduction in *Bm-cpl* transcript level (Fig. 6). Analysis by qRT-PCR on RNA isolated from the RNAi treated worms, which had identical phenotypes to those shown in Figs. 1&3, showed that the *Bm-cpl* transcript levels were reduced by 51.0% in *Bm-cpl-5* dsRNA treated worms and by 48.9% in the siRNA treated worms, these reductions were significantly different from both the medium control (dsRNA; *P* = 0.0006, siRNA; *P*<0.0001) and the negative control (dsRNA; *P* = 0.0033, siRNA; *P*<0.0001). The *Bm-cpl* transcript levels were also reduced in *Bm-cpl Pro* treated worms; 66.5% and 37.4% in dsRNA and siRNA treated worms respectively, with significant differences from both the medium control (dsRNA; *P* = 0.0003, siRNA; *P* = 0.002) and the negative control (dsRNA; *P* = 0.0016, siRNA; *P* = 0.0082). The percent reduction was normalized using a tubulin (*Bm-tub-1*) transcript. In comparison, the *Bm-cpl-3* (89% identical to *Bm-cpl-2* and *-7*) and *Bm-cpl-6* transcripts that belong to the Ic subgroup of the *B. malayi* cysteine protease protein family were not reduced in the *Bm-cpl Pro* treated worms (data not shown). These data demonstrate that the inhibition of Mf secretion *in vitro*, death of Mf, and changes in intrauterine progeny following dsRNA-mediated silencing is likely associated with reductions in the *Bm-cpl* gene specific transcript levels belonging to group Ia.

## Discussion

Cysteine proteases play important roles in both intracellular and extracellular processes which are important in both development and survival. Cathepsin-like enzymes have been identified as potential targets for drug or vaccine development in many parasites, including filarial nematodes [33],[38], due to as their potential essential roles in feeding [46],[47] [48], molting [33],[49], embryogenesis [40],[44] and immune functions [50] (reviewed in [51]). Here we describe the use of RNAi techniques in *B. malayi* adult females to directly assess the function(s) of the cysteine protease (CP) genes that belong to the CPZ and the group Ia of *Bm*-CPL protein families.

In filarial nematodes the gene family of group Ia cathepsin L-like protease enzymes (*Bm-cpl*: *Bm-cpl-1*, *Bm-cpl-4* and *Bm-cpl-5*) has been shown to be associated with larval molting and remodeling of the cuticle and eggshell [39]. Antibodies raised to *Ov*-CPL-1 (64–65% identity with *Bm*-CPL-1, -4 and -5) and *Bp*-CPL-4 (77, 91 and 94% identity with *Bm*-CPL-1, -5 and -4, respectively) recognized the reproductive system in *B. malayi* similarly [39] (S. Lustigman, unpublished data), and this localization was similar to that of *Ce*-CPL-1 [40]. The putative functions of the group Ic cathepsin L-like protease enzymes (*Bm-cpl-2*, *Bm-cpl-3*, *Bm-cpl-6*, *Bm-cpl-7* and *Bm-cpl-8*) are still unknown. In *C. elegans*, *Ce-cpl-1* was shown to be essential for embryogenesis [40], while *Ce-cpz-1* has a function during embryogenesis but is not essential and also has an important role during molting [44]. The *C. elegans Ce-cpl-1* knock-out mutant has an embryonic lethal phenotype [40]. RNAi targeting of *Ce-cpl-1* produced an early embryonic lethal phenotype with 95–100% of F_1_ embryos arresting with only 100–200 cells following dsRNA injection and ∼92% of F_1_ embryos arresting after soaking of L4 in dsRNA [40]. Interestingly, the *H. contortus cpl-1* gene can rescue the *C. elegans cpl-1* RNAi effect, suggesting that the parasite CPL is an orthologue of *C. elegans* CPL-1 and that CPL-1 is functionally conserved in parasitic nematode species [52]. This is further demonstrated in *O. volvulus*, where *Ov-cpl-1* can rescue the *C. elegans Ce-cpl-1* mutant, using a rescue construct containing the *Ce-cpl-1* promoter, the full length *Ov-cpl-1* cDNA from the first ATG to the stop codon and the *Ce-cpl-1* 3′UTR (S. Hashmi, unpublished data).

However, despite high sequence similarity the predicted essential function of a gene is not always essentially conserved in both filarial nematodes and *C. elegans*, as has been shown for the different roles of CPL and CPZ in the molting of *O. volvulus* and *C. elegans* [33]. dsRNA-mediated silencing of the *Bm-cpl* group Ia genes in *B. malayi* adult females leads to phenotypic changes in embryos, where embryos from *Bm-cpl* treated worms were not fully developed within the eggshell, leading to space between the embryo and eggshell. Embryonic viability following RNAi treatment with *Bm-cpl* was also affected.

The role(s) of cysteine proteases during embryo development have been shown to include an essential role in yolk processing. Cysteine proteases are known to be involved in yolk degradation during invertebrate embryonic development [53],[54],[55] and *Ce*-CPL-1 has been shown to play an essential role in yolk protein processing during embryonic development, where a loss of CPL-1 activity in the *Ce-cpl-1* mutant leads to formation of enlarged cytoplasmic yolk vesicles and embryonic lethality [41]. Whether the *Bm-cpl* cysteine proteases are also involved with yolk protein processing would be interesting to investigate. Immunolocalization of the native *Bm*-CPLs indirectly suggests that they do localize to yolk vesicles within the developing embryonic stages [39] but further co-localization studies are required to confirm these observations. Also of relevance would be to investigate the co-localization of the native *Bm*-CPLs with their endogenous cysteine protease inhibitors, such as the cystatins [56]. The activities of cathepsin-like cysteine proteases are controlled by their specific endogenous protein inhibitors [57],[58]. In *C. elegans*, co-localization of CPL-1 and CPZ-1 enzymes with both endogenous CP inhibitors and yolk proteins has been clearly demonstrated and data indicates that CPL-1 and CPZ-1 and their putative inhibitor, *Ce*-CPI-2a, play a role during oogenesis and fertilization in *C. elegans* [59]. The *O. volvulus* CPL-1 and CPZ are also localized in the same regions as the endogenous inhibitor, *Ov*-CPI-2 [40],[49], implying that *Ov*-CPI-2 may regulate both enzymes during *O. volvulus* development. In addition, both enzymes are essential for third to fourth stage larva molting as demonstrated by RNAi [33].

dsRNA-mediated silencing of the *Bm-cpl* genes suggests that some of the *B. malayi* CPs such as the *Bm*-CPL group Ia enzymes may function during embryonic development and show similar function(s) to *C. elegans* CPL-1, their most phylogenetically related CP, therefore suggesting conserved essential function(s) between these filarial nematode and *C. elegans* CPs. As CP inhibitors targeting parasite CPs have been proposed as therapeutics in both protozoan [60],[61],[62],[63] and metazoan [64],[65] parasites, CPs involved in embryogenesis clearly provide a valuable putative target if it results in a block in embryogenesis which, in filarial nematodes, would, in essence, lead to sterility of the adult worm. Therefore, it would be invaluable to determine specific cysteine protease inhibitors which would target the endogenous *Bm*-CPL group Ia enzymes and moreover, could be used as therapeutic agents against the parasite. Methodologies, such as synthetic combinatorial library analysis [66], specific chemical library screening [67] and small molecule affinity fingerprinting [68] are available to identify such specific inhibitors.

In order to elucidate the function(s) of the cysteine proteases of *B. malayi* we have developed and improved the reverse genetics RNAi techniques which have been used previously in filarial nematodes. RNAi has been demonstrated successfully in parasitic helminths and could provide an invaluable tool in determining gene function and identification of drug and vaccine targets. Using RNAi to selectively target the potential drug targets; the cathepsin-like cysteine protease genes, in adult female *B. malayi* we have demonstrated that these genes can be affected; dsRNA-mediated silencing of the *Bm-cpl* genes led to significant reductions in the number of Mf released by adult female nematodes. While *Bm-cpl-1*, *Bm-cpl-5* and *Bm-cpz* all showed this persistent phenotype following dsRNA-mediated silencing the most significant reductions in the release of Mf were observed with dsRNA corresponding to *Bm-cpl-5* therefore we focused on optimization of the RNAi technique using fragments corresponding to *Bm-cpl-5* and the 100% conserved pro-region of *Bm-cpl-1*, *Bm-cpl-4* and *Bm-cpl-5*, designed to target all three of the *B. malayi* filarial group Ia cathepsin L-like cysteine protease transcripts [39], which we named *Bm-cpl*. Treatment of adult worms with dsRNA or siRNA corresponding to the *Bm-cpl* genes resulted in a persistent and significant reduction in the release of Mf, the majority of which were dead, and changes in intrauterine progeny. At the same time, these treatments also resulted in a specific reduction in the *Bm-cpl* transcript levels. While we can not directly correlate the observed phenotypic effects with the specific reduction in *Bm-cpl* transcript levels it is tempting to speculate that the effects on embryonic development and Mf production are due to the specific knock-down of the *Bm-cpl* genes using the dsRNA-silencing pathway.

Interestingly, while RNAi treatment using both dsRNA and siRNA were effective in specific gene silencing, the uptake studies have suggested that smaller fragments of dsRNA and siRNAs are able to penetrate adult female *B. malayi* worms more efficiently than longer dsRNAs, pointing to the possibility that the cuticle barrier in the female worm is more yielding to the penetration of smaller fragments of dsRNA and siRNA. Therefore, the efficiency of RNAi may be dependent on the dsRNA fragment size. It is also important to note that due to RNA fragment size differences the molarity of each dsRNA tested was different (dsRNA range; 400–800 bp, siRNA; ∼13.5 bp, at 1.5 mg/ml; dsRNA range; 2.82–5.63 µM, siRNA; 166.83 µM, at 2.0 mg/ml; dsRNA range; 3.75–7.51 µM, siRNA; 222.44 µM). However, despite these molar differences, RNAi using longer dsRNA was still effective in this *in vitro* screening system. dsRNA molarity has been shown to affect the efficiency of RNAi targeting of actin in *L. sigmodontis* (*Ls-act*) with 3.5 µM giving the most consistent reductions in transcript, while higher molar concentrations of dsRNA (17.5 and 35 µM) resulted in increased levels of heat shock protein 60 (*Ls-hsp60*) suggesting the worms were stressed [35]. Therefore dsRNA molarity might still be an important consideration to keep in mind when optimizing RNAi techniques.

While successful siRNA application has been reported in *T. colubriformis* [69] and *S. mansoni* [70],[71] it has not previously been documented for filarial nematodes. We have shown for the first time that RNAi treatment using siRNA can also be effective in filarial nematodes and show the same phenotypic changes and reduction in transcript levels as RNAi treatment with fragments corresponding to the same region of dsRNA. This provides an additional RNAi strategy for future projects aimed at assessing the function(s) of genes in filarial worms and the discovery of novel drug targets. This is important as many of the inconsistencies with RNAi in parasitic helminths could be due to differences in the RNAi pathways between these helminths and free-living *C. elegans*. Analyses of genome databases, including *H. contortus* [30] and *B. malayi* [72], have shown that while putative orthologues of many genes required for RNAi are present, other genes which are essential for the recognition and systemic spread of dsRNA in *C. elegans*, such as *rde-4*, *sid* and *rsd* genes, appear to be absent. The absence of these genes could explain the problems associated with RNAi in parasitic helminths, although it could be that these genes are not required or alternative pathways exist. However, although the genes for dsRNA processing appear to be missing, the machinery for processing siRNA appears to be present. Therefore, the use of siRNA vs. dsRNA may provide a more robust RNAi assay for some of the target transcripts.

We have also tested an alternative method for delivering the dsRNA molecules to adult *B. malayi*, which are notoriously difficult to maintain in culture for extended periods [73], by culturing worms in dialysis tubes in the presence of dsRNA. This technique provides a way of minimizing the handling of nematodes and has the potential to facilitate higher-throughput RNAi screens. Dialysis of the dsRNA prior to RNAi treatment was also important in reducing the non-specific off-target effects which can occur following RNAi [74]. In filarial parasites treatment with control dsRNA, resulted in a 24.7–49.8% reduction in molting of *O. volvulus* L3 larvae [32],[33], and resulted in reduced motility in *B. malayi* adult worms [34]. Off-target effects following RNAi treatment have also been demonstrated in *S. mansoni*, these including non-specific changes in mRNA levels, changes in cercariae distribution and reduction in sporocyst length following treatment with control dsRNA [75],[76]. We noted some non-specific toxic effects of unrelated dsRNA; for example the RNAi with the negative control dsRNA we selected to use, *Ov-cpz-int2*, resulted in 14.9% reduction in the release of Mf 48 h after RNAi. However, this was not accompanied by any indirect knock-down in target gene transcription. *E. coli* β-lactamase (*Ec-bla*) dsRNA (GenBank accession no. NC_010862, position 24517-24770) [43] was also used as a negative control and also showed some off target effects (1.8%, 10.5% and 26.0% reduction in Mf release on day 1, 3 and 5). Regardless, the effect with the gene specific RNAi was significant. These off-target effects could, in part, be due to an innate immune response mounted in the nematode in response to foreign RNA. Viral RNAs are recognized by the innate immune response through toll-like receptor (TLR) 3 for dsRNA and TLR7 or TLR8 for ssRNA [77]. In addition, RNA silencing serves as an innate anti-viral mechanism in plants, fungi and animals [78],[79], and has been shown to be involved in *C. elegans* [80]. RNAi silencing in nematodes may have evolved to protect them against viruses and it could be speculated as a reason to why nematodes appear to be free from viruses. Another possibility is that the off-target effects could be due to the generation of microRNAs (miRNAs) from the dsRNA control sequence used. Intronic sequences have been shown to potentially also contain functional miRNAs [81],[82]. However, searching the intron sequence used in this study (*Ov-cpz-int2*) shows that it contains no miRNA regions so this appears unlikely.

In order to effectively assess the essential function(s) of filarial genes using RNAi it is important to keep in mind the unpredictability associated with RNAi and to take care to optimize the conditions according to the target gene of interest. Suppression of gene expression following RNAi treatment, using current methodologies, appears to be more effective for some genes rather than others [83],[84]. This could be due to experimental parameters such as dsRNA preparations, length of dsRNA, and regions of dsRNA amplified, or parasite factors including abundance and location of the target gene transcript. As seen in *C. elegans* the life-cycle stage may also be important; RNAi is often not efficient in the treated worms (P_0_), and the phenotypic effects are only obvious in the progeny (F_1_), after the target mRNA that is produced in the F_1_ generation is also degraded [85]. Our studies, unfortunately, can only measure the phenotypic and genotypic outcomes in treated (P_0_) adult female worms. When the steady-state transcript levels in the adult worms are high it can be difficult to observe significant reduction even though the phenotypic release of F_1_ progeny is affected. One potential solution will be, in the future, to also measure the transcript levels of the targeted mRNA in the F_1_ progeny within the uterus or secreted into culture. In order to be able to obtain information as similar as possible to *C. elegans* the culture conditions in parasitic nematodes need to be optimized in order to maintain parasites for longer periods of time and, ideally, to allow for their subsequent development into the next life-cycle stages. The effects of RNAi treatment may also need to be studied directly *in vivo* or after monitoring the development of the F_1_ following treatment of adult worms *in vitro* then passing the secreted Mf through the full life-cycle *in vivo*. It may also be possible to perform RNAi directly on early developmental stages such as eggs and embryos. Using uptake studies we can demonstrate that dsRNA is taken up into eggs and embryos isolated from the uterine contents of adult female *B. malayi* (L. Ford&S. Lustigman, unpublished data).

In order to maximally utilize RNAi in parasitic helminths as a tool for determining gene function and for high-throughput screening of potential drug and vaccine candidates, various problems need to be addressed and optimization of both the long-term culturing conditions and RNAi techniques is required. By modifying both ours and published RNAi techniques we have shown persistent dsRNA-mediated silencing and demonstrated improved RNAi techniques.

In conclusion, RNAi assays have allowed us to examine the function(s) of the cathepsin-like cysteine protease filarial group Ia genes in *B. malayi* adult female worms and have demonstrated that they have function(s) in embryogenesis and development which are similar to their orthologous genes in *C. elegans*. Thus, validating the potential of the filarial cysteine proteases as promising drug targets for filarial chemotherapy.
